# Digital surface enhanced Raman spectroscopy for quantifiable single molecule detection in flow†

**DOI:** 10.1039/d4an00801d

**Published:** 2024-06-13

**Authors:** Hannah C. Schorr, Zachary D. Schultz

**Affiliations:** a Department of Chemistry and Biochemistry, The Ohio State University Columbus OH 43210 USA schultz.133@osu.edu

## Abstract

Surface enhanced Raman scattering (SERS) provides a label free method of analyzing molecules from diverse and complex signals, potentially with single molecule sensitivity. The chemical specificity inherent in the SERS spectrum can identify molecules; however signal variability arising from the diversity of plasmonic environments can limit quantification, particularly at low concentrations. Here we show that digitizing, or counting SERS events, can decrease the limit of detection in flowing solutions enabling quantification of single molecules. By using multivariate curve resolution and establishing a score threshold, each individual spectrum can be classified as containing an event or not. This binary “yes/no” can then be quantified, and a linear region can be established. This method was shown to lower the limit of detection to the lowest physical limit, and lowered the limit of detection by an order of magnitude from the traditional, intensity based LOD calculations.

## Abstract 2

Event counting at low concentrations enables the quantitative surface enhanced Raman scattering detection of single molecules in flowing solutions.

## Graphical abstract

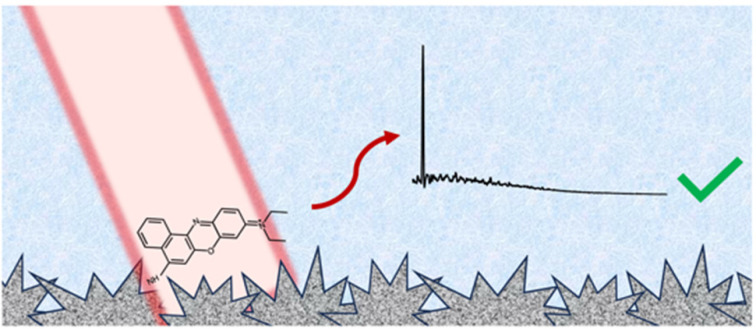

Single molecule detection with surface enhanced Raman spectroscopy (SERS) is possible,^1–3^ but difficult to achieve in practical applications, such as in online detection with flowing solutions.^4^ The ability to count single molecules in flow offers tremendous potential for detecting trace biomarkers in complex biological samples. The ability to lower the limit of detection and quantify such trace markers can improve studies in metabolomics, proteomics, or even in chemical reaction monitoring. Many analytes, such as metabolites for example, are present at very low concentrations in living cells,^5^ so expanding the lower limit of the linear dynamic range of SERS can expand the method to more applications. Biological samples also tend to be complex mixtures, so label free detection in static solution proves to be difficult. By combining SERS with a separation method, such as high performance liquid chromatography or capillary electrophoresis, label free detection in flow becomes even more feasible and useful.

Single molecule detection in flow is useful for very low concentration analytes, but often requires the use of a reporter molecule. Current methods in single molecule detection are mostly fluorescence based, and tend to require binding to an antibody or a fluorescent tag before single event counting can take place.^6–8^ Therefore, a need exists for a label free, single molecule counting method that can be applied in flow.

It is well known that SERS signals are very bright, even when they occur from only one molecule. Signal averaging, whether through long acquisition times or through post-processing, can actually cause the loss of this valuable information. If one spectrum out of a thousand shows a signal, then it is likely to be averaged out, and that set of data would be assumed to show “no signal”.^9^ One way to expand the linear dynamic range of SERS is to digitize the collected signals. By collecting spectra on the relative timescale of molecular diffusion^10^ and counting stochastic events in samples with low concentrations of analyte, it is possible to bridge the gap between the frequency, or counting domain, and the intensity, or ensemble domain, and thus lower detection limits.

Digitizing SERS signals is an approach that has been successfully demonstrated on static samples.^11,12^ Imagine rastering a laser across the surface of a planar substrate. In very low concentration regimes, the laser spot will only illuminate one analyte molecule in a hot spot at a time. In this case, while the intensity may vary due to the enhancement of each hotspot, a single molecule has most probably been excited, so it is possible to count the signal events that occur. In higher concentration regimes, however, the laser likely illuminates several analyte molecules at once. The intensity of the signal will then scale approximately with concentration. This is the ensemble regime in which experiments are typically performed.^11,12^

In flow based SERS detection, it is useful to use a planar substrate to produce more consistent signals and remove the chance of random nanoparticle aggregation.^9,13^ In this case, a laser is not rastered across a sample illuminating different hot spots and different nanoparticles at each spot. Instead, the laser stays stationary and illuminates the same hotspot or collection of hotspots while the analyte flows through the laser spot. Detection, then, requires close interaction between the analyte and the nanostructured substrate. Due to the distance that an analyte must diffuse, this is a low probability event. The probability can be increased using hydrodynamic focusing, or sheath flow, to promote the interaction between an analyte and a planar substrate.^14–16^ With a sheath flow cell, a faster moving sheath fluid is flowed over the slower moving analyte stream. This creates a confinement effect that limits the distance that an analyte molecule must diffuse to interact with the substrate, thus enabling more efficient detection and lowering detection limits. The sheath flow also aids in surface regeneration by simply turning off the sample flow and allowing analyte molecules to wash away. Illustrations of the sheath flow cell used and how sheath flow confines an analyte to the substrate surface are shown in Fig. S1.†

Here we present results illustrating proof-of-concept that digitizing the SERS signal from a model SERS analyte can extend the useful concentration range for quantifying an analyte in a flowing solution. To demonstrate this, we have used our previously reported and characterized sheath-flow SERS setup that incorporates sheath-flow detection with the custom SERS spectrometer.^5,14–22^ Interestingly, in low concentration capillary electrophoresis experiments with this SERS detection scheme, an extreme band narrowing was observed.^14^ This extreme apparent resolution appeared to arise from detection of individual Rhodamine isomers migrating out of the separation capillary within concentrated and narrow bands. These observations suggested single molecule quantification may be possible.

Pattern recognition to uniquely identify the SERS response from a molecule are important for accurate analysis. Chemometric methods can be utilized for digitizing SERS signals. Multivariate curve resolution (MCR) was used here. MCR is a bilinear data reduction method that provides both spectral profiles (loadings) and quantitative concentrations (scores).^23,24^ The scores describe how well a particular data point matches with a loading. In this way, it can be assured that the whole spectrum is being considered and not just a single peak in the spectrum. This is especially helpful for more complex spectra, or spectra with peaks that are difficult to resolve.^13,23^ Using MCR also ensures that the spectral components are being fit, and not just random fluctuations in the noise of each spectrum.

Towards the goal of decreasing the limit of detection to one molecule, the traditional limit of detection determined first. Nile Blue A (NBA) was chosen as a model analyte due to its relatively large Raman scattering cross section and its absorption maximum of 630 nm, which could allow for resonance Raman effects as well.^25,26^ Because NBA is on resonance with the 632.8 nm HeNe laser being used for the Raman measurements, at higher concentrations a fluorescence background becomes an issue.^27^ The SERS spectra of each concentration of NBA was recorded in three separate experiments. To remove the fluorescence background and prevent it from being an issue in chemometric analysis, a rolling circle filter was used to background correct each spectrum.^28^ This results in spectra with flat backgrounds, but it does not remove SERS spectral details. Background corrected spectra showing the average of all acquisitions can be found in Fig. 1, while uncorrected average spectra are shown in Fig. S2.†

**Fig. 1:**
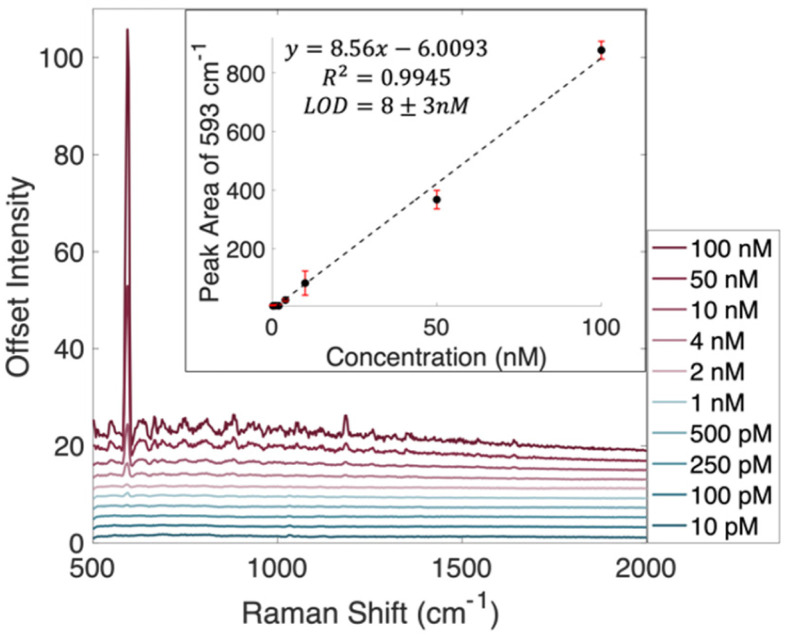
Background subtracted spectra of Nile Blue A at concentrations ranging from 10 pM to 100 nM. Inset plot shows calibration curve created using the area of the peak at 593 cm^−1^. A limit of detection of 8 ± 3 nM was calculated using three times the standard error in *y* over the slope of the line of best fit. Error bars are the standard deviation from triplicate measurements.

The inset plot in Fig. 1 shows the calibration curve created using the linear region of the peak areas at 593 cm^−1^. Using this traditional, intensity based method, a limit of detection was found to be 8 ± 3 nM. This LOD was calculated using 3 times the standard error in y divided by the slope of the line of best fit.

Using the baseline corrected spectra, a multivariate curve resolution (MCR) model was calculated. To ensure that variance captured by the first component correlated to the Nile Blue A spectrum, the model was trained using the average spectrum from the 100 nM samples. This prevented confusion in the model arising from varying noise profiles in multiple acquisitions and low signal experiments. All other collected spectra were loaded into the model as a validation set. Fig. 2 shows the distribution of scores for each concentration. The scores were then analyzed to determine what was considered an event and what was not. The dashed line in Fig. 2 denotes the event threshold. This threshold was calculated using three times the standard deviation of the scores of the blank (water) plus the average score of the blank. Anything that falls above this range is classified as a countable signal.

**Fig. 2:**
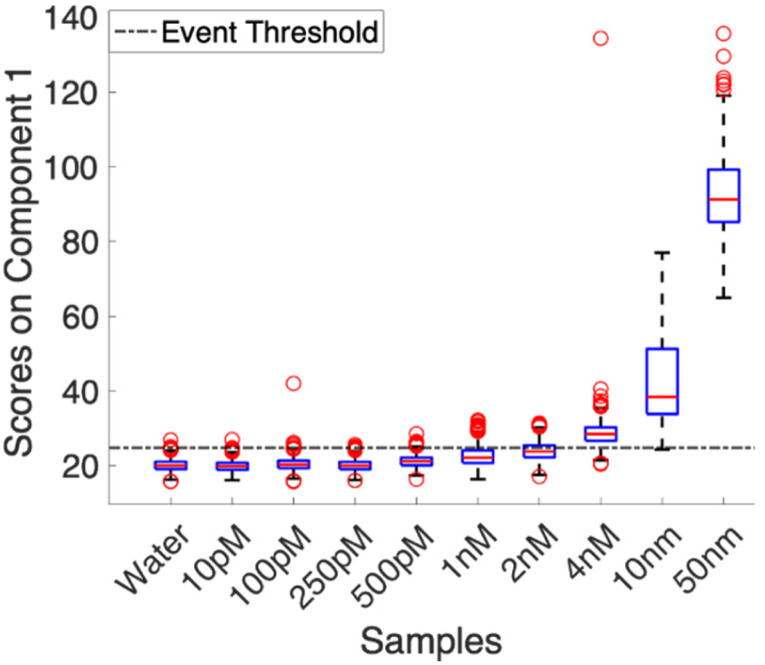
Distribution of scores for each sample concentration. The dashed line shows the event threshold (24.8), which was calculated using the three times the standard deviation of the scores of the blank. Error bars are the standard deviation from triplicate measurements.

Examining the raw data shows spectra with features clearly detected at concentrations below the calculated LOD. Defining spectra with MCR scores above the threshold shown in Fig. 2 provides a means to quantify these occurrences. The prevalence of these events is observed to increase with concentration. Fig. 3 shows the percentage of spectra that counted as events for each concentration. The general trend shows a Langmuir type fit, which is expected, as at a certain point all spectra in a series will contain signal, and the signal will start scaling with intensity. The inset plot shows the linear region of this fit. The limit of detection was then calculated as 500 ± 200 pM using 3 times the standard error in y over the slope of the line of best fit. This is a 16 fold, more than an order of magnitude, decrease from the LOD calculated using traditional intensity scaling.

**Fig. 3:**
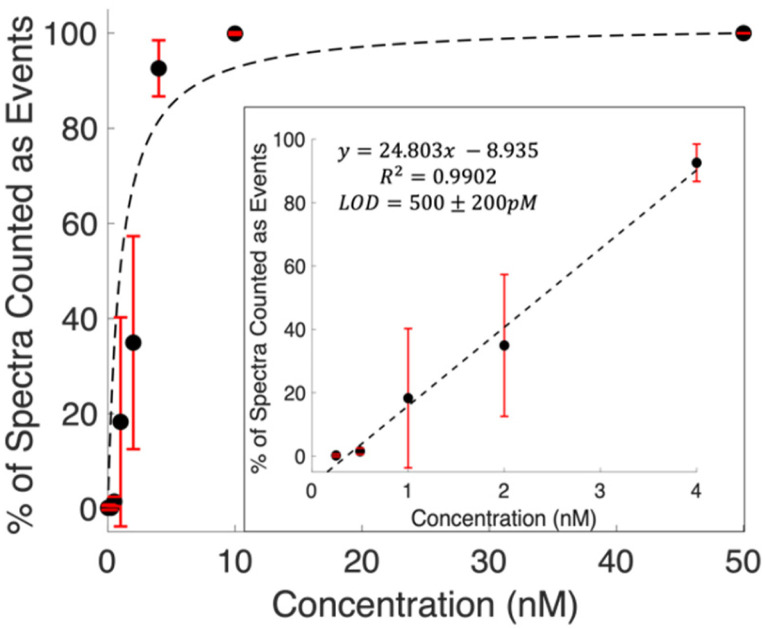
Curve showing the percent of spectra from each concentration that are considered to be events. The curve levels off to 100% around 10 nM. The inset plot shows the linear region of this curve. A digital limit of detection of 500 ± 200 pM was calculated using 3 times the standard error in *y* over the slope of the line of best fit. Error bars are the standard deviation from triplicate measurements.

Examples of individual spectra that scored as an event, and a spectrum that did not, are shown in Fig. S3.† Analysis of consecutive spectra, as shown in Fig. S4,† show that signals can persist for a single frame, 10 ms, and go away, demonstrating regeneration of the surface and non-permanent interactions with the nanostructures. While the signal to noise on these spectra is relatively low due to the 10 ms acquisition time, the MCR model was able to pull out spectra that contain expected features. Most notably, the strong feature at 593 cm^−1^ is present, even faintly, in all of the “yes” spectra, and absent in the “no” example. For spectra that were classified as a “yes” in the single molecule counting regime, most scored around 27.5 ± 2.5 on the MCR model. This consistency in score supports that we are most likely investigating single molecules in the same hot spots. While there is some variability, it is most likely coming from differences in orientation and different levels of interaction with the nanostructures on the surface. This is also an advantage of utilizing a planar substrate as opposed to a colloidal solution as the same hotspots will be illuminated while the solution is in flow, and there is no risk of random aggregation. This provides consistent, repeatable signals. In the higher concentrations, when the data presents as an ensemble measurement, scores begin to vary widely and generally increase, indicating that there are more molecules in the hot spot at a time, and that the ability for single molecule counting has been lost.

The ability to count individual molecules by SERS in flow is already at a disadvantage compared to static methods as the analyte is moving at a particular velocity and must interact with a hotspot on a planar substrate while in the laser spot. The low probability of these events aligning leads to detection limits that are higher than those of static methods. Detection limits are further limited by signal averaging. Though SERS events are bright, a singular event would be washed out by long acquisition times or by averaging the one spectrum that shows signal with many that only contain noise. The sheath flow interface previously reported^29^ increases interaction between the analyte and the SERS substrate, and acquisition times that match the approximate diffusion time for molecules are used. By lowering acquisition times and considering each collected spectrum, the SERS events are able to be distinguished and digitized, lowering the limit of detection.

The analyte flux, the number of molecules present in the flow cell at a time can be calculated as follows:1
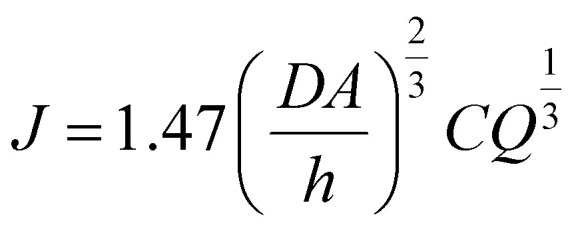
where *J* is the flux to the surface, *D* is the diffusion coefficient of the analyte, *A* is the area of the surface, or in this case the area of the laser spot, *h* is the height of the channel, *C* is analyte concentration, and *Q* is volumetric flow rate.^30^ Using eqn (1), it is calculated that there are approximately 2 molecules in the laser volume during the time of each acquisition at the lowest concentration. This calculation supports the sparsity condition required for proving single molecule detection.^11,31^ It is not guaranteed, however, that these molecules are interacting with the surface of the nanostructured substrate as this value is calculated using the entire height of the channel. While sheath flow helps promote interaction with the surface, there is still a significant layer thickness formed over the laser spot on the SERS substrate. Further information on this calculation can be found in the ESI.†

Digitizing the SERS signals takes advantage of single molecule events, or single interactions in a hot spot in the laser spot at a time. Although we were able to detect single molecules of NBA, a model SERS analyte, some molecules that are less ideal for SERS detection may require higher concentrations and may not be detectable at picomolar concentrations. What this method is really taking advantage of and is hoping to achieve is to detect the concentration where single events are occurring for that particular analyte. For something like glucose, which has a high SERS limit of detection due to a lower Raman scattering cross section and a repulsion towards metals, lowering the limit of detection for SERS might look like pushing the LOD to micromolar concentrations when the traditional intensity based LOD is in the millimolar concentration.^32–34^

## Conclusions

Applying a digital counting method to SERS in flow enables decreased limits of detection and quantification of Nile Blue A. Interactions of dilute solutions with the SERS substrate are increased using a sheath flow SERS cell. Using MCR, the spectrum of the target analyte is matched and used to count the number of molecules interacting with the surface. A calibration curve was created for the number of digitized events showing a limit of detection of 500 pM, which is an order of magnitude lower than the traditional, intensity based detection limit of 8 nM. This result shows that it is possible to quantify below the standard limits of detection by taking advantage of individual, bright spectra, single molecule detection, and multivariate analysis in flowing sample solutions. This holds further promise for chemical specific, quantifiable detection in chromatographic or other solution phase experiments.

## Data availability

The data supporting this article have been included as part of the ESI.† Requests for data in a digital format (*e.g.* .csv) are available upon request.

## Conflicts of interest

There are no conflicts to declare.

## Supplementary Material

AN-149-D4AN00801D-s001
