# Supplementary material for: Digital surface enhanced Raman spectroscopy for quantifiable single molecule detection in flow
Source: Analyst. 2024 Jun 13;149(14):3711–5. doi: 10.1039/d4an00801d (PMC11229883; doi:10.1039/d4an00801d)
Supplement: AN-149-D4AN00801D-s001 [file AN-149-D4AN00801D-s001.pdf]

Electronic Supplementary Material (ESI) for Analyst.
This journal is © The Royal Society of Chemistry 2024

Electronic Supporting Information: Digital surface enhanced Raman spectroscopy for quantifiable single molecule detection in flow

Hannah C. Schorr and Zachary D. Schultz*
Department of Chemistry and Biochemistry, The Ohio State University, Columbus, OH 43210

*Corresponding author email: Schultz.133@osu.edu

Supplementary Information

Contents

Contents 1
Methods 1
Materials and Reagents 1
SERS Substrate Preparation 1
Raman Detection 2
Figure S1: Schematic representation of sheath flow 2
Data Analysis 3
Results 3
Figure S2: Raw average SERS spectra of Nile Blue A 3
Figure S3: Examples of "yes" and "no" spectra from various runs 4
Figure S4: Consecutive spectra showing "yes" and "no" examples 4
Equation 1 4
References 5

Methods

Materials and Reagents
Nile Blue A (dye content >75%) and sodium hydroxide pellets (≥ 97%, ACS Reagent) were purchased from Sigma Aldrich (St. Louis, MO). Naonpure water (18.2 MΩ) was obtained from a Thermo-Scientific Genpure system. Bare fused silica capillary with 150 µm outer diameter and 75 µm inner diameter was purchased from Polymicro Technologies (Phoenix, AZ).

SERS Substrate Preparation
Thermally evaporated SERS substrates were prepared as previously reported.[1] Briefly, Silver pellets (Kurt J. Lesker Company, Jefferson Hills, PA) were evaporated into 13 mm diameter (linear surface area 530 mm^2) anodized aluminum oxide (AAO) filter with 0.2 µm pores (Global

Life Sciences, Buckinghamshire, UK). This substrate was then soaked in 0.1 M sodium hydroxide for 4 hours to remove the AAO and reveal a heterogeneous, nanostructured Ag surface. The substrates were stored under vacuum following thermal evaporation and were not soaked in sodium hydroxide until use. Using this method, substrates remain stable for at least 3 months of storage. These substrates have been shown to have an average surface enhancement factor of 0.6 x 10^8 and a relative standard deviation across the surface of less than 20%.[1]

Raman Detection

A homebuilt Raman spectrometer was used to collect in flow measurements. A 632.8 nm HeNe laser at 1 mW was focused onto a substrate in a flow cell previously described by the Schultz lab.[2, 3] Briefly, a plastic base plate has been fitted with both an inlet and and outlet port to facilitate a sheath flow. A silicone gasket is placed atop the base plate, and a glass slide that has been drilled with holes to accommodate the sheath flow ports is next. A prepared substrates sits atop the slide and is affixed with clear nail polish (LA colors, Ontario, CA). A 75 uM inner diameter capillary, with the outer coating burned off of the first approximately 10 mm, is laid across the top of the substrate. A 2 mm wide flow channel is defined using a 500 µm thick silicone gasket. A cover slip is placed on top of the gasket, and the flow cell is closed by screwing a stainless steel top plate down to create a seal. A 40x, NA = 0.8, water immersion objective from Olympus was used for focusing the laser and collecting Raman scattering. The Raman scattered light was then directed to an Andor Shamrock 303i spectrograph and then to an Andor iDus 401 CCD. Raman spectra (1000+ per sample per trial) were recorded in series with 10 ms acquisition times. Concentrations of Nile Blue A ranging from 10 pM to 100 nM in aqueous solution were each analyzed in triplicate. This homebuilt spectrometer has a similar sensitivity to commercial Raman spectrometers such as the Renishaw Qontor inVia Raman microscope and a benchtop Snowy Range benchtop Raman spectrometer. This was validated by a standard calibration curve of ethanol, where slopes of the normalized calibration curves are nominally similar, and all three instruments shows LODs of 20-40 mM.[4] The flow rates used in the flow cell were 1 µL/min for the sample and 30 µL/min for the sheath fluid. The sheath fluid used in these experiments was water. The flow cell can be visualized in Figure S1A, and the effects of sheath flow are shown in Figure S1B and S1C.

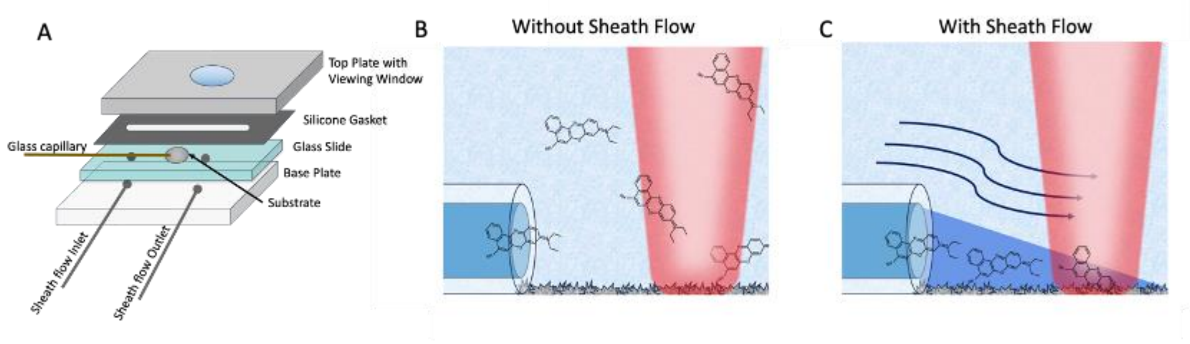

Figure S1: Schematic illustrating sheath flow. A. Illustration of the sheath flow cell. Starting from the bottom up, a FEP base plate is fitted with an inlet and outlet port for the sheath flow. A glass slide that has had inlet and outlet ports drilled through it is

placed on the base plate. The silver substrate is prepared on this slide, and a glass capillary is laid across the slide and the substrate. The channel of the flow cell is defined with a silicone gasket. A cover slip and a stainless steel top plate are placed on the top, and the whole flow cell is screwed together (screws not pictured). B. Illustration of a cross section of the flow channel if no sheath flow is applied. Molecules are free to diffuse to the whole height of the channel and may not interact with the substrate. C. Illustration of a cross section of the flow channel if sheath flow is applied. The analyte stream is confined closer to the bottom of the channel, and thus analyte molecules have a better chance at interacting with the substrate. When two fluids are introduced to each other in a laminar flow regime, such as in a microfluidic channel, they will continue to flow side by side. If the two streams are moving at the same velocity, they would occupy the same amount of space each. As the sheath fluid is moving at a higher velocity, it is exerting more pressure on the slower moving analyte stream and must take up more space in the channel. This causes the analyte stream to compress to one side of the channel, in this case, the bottom, where the substrate resides.[3, 5-8]

Data Analysis

All spectral data was processed in Matlab 2020a (Mathworks). Peak fitting was performed using the code Peakfit.m, available publicly for Matlab.[9] Spectral peaks were fit to a gaussian line shape. Prior to any analysis, the spectra were background corrected using a rolling circle filter with a radius of 500.[10] Multivariate curve resolution was performed using the PLS Toolbox (Eigenvector Research, Inc.) operating in Matlab.

Results

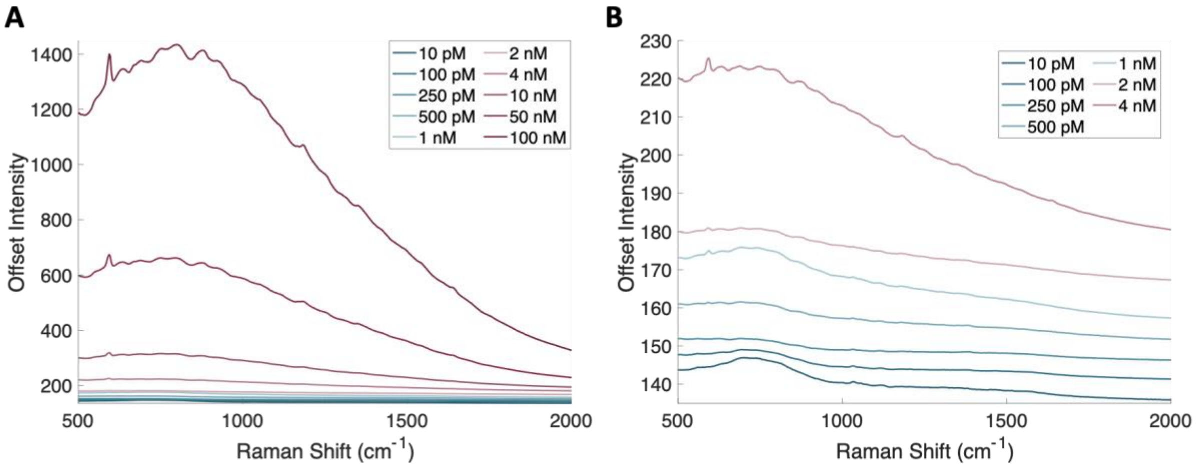

Figure S2*: (A) Average SERS spectra of Nile Blue A at various concentrations. (B) The same SERS spectra of Nile Blue A on a different Y-scale to showcase lower concentration spectra.*

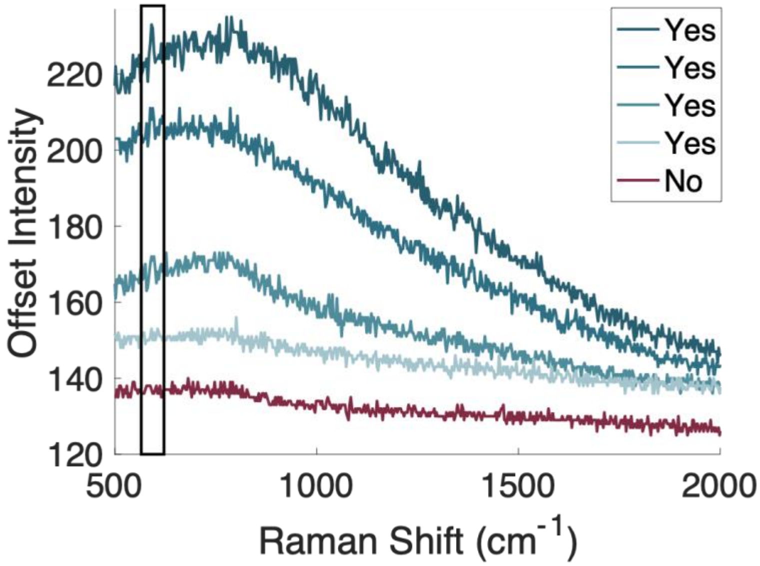

Figure S3: Individual spectra from runs. Spectra in blue show events that scored over the threshold value on the MCR model. Spectrum in red did not score above threshold value on MCR model. Spectra that scored above the threshold seem to have the expected feature at 593 cm^{-1}, highlighted in the black box.

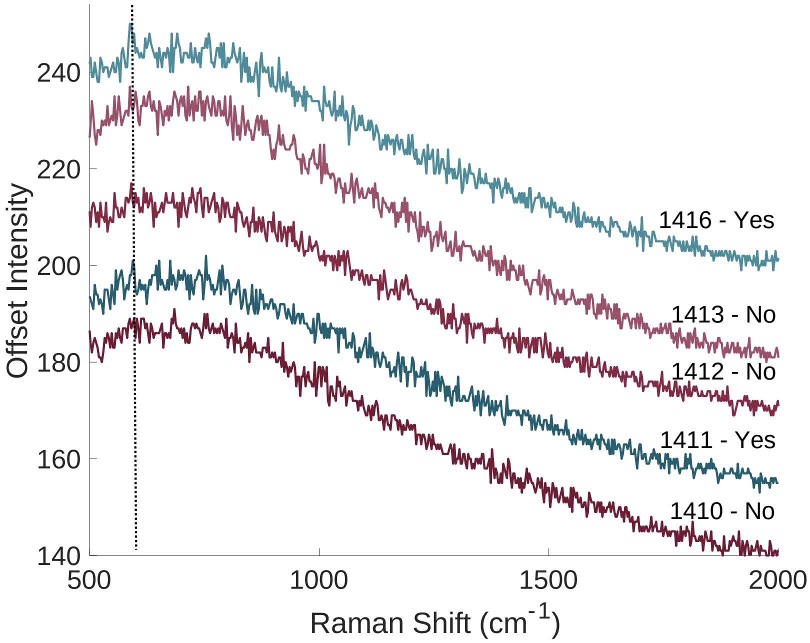

Figure S4: Individual consecutive spectra from the 2 nM collection. Spectrum number indicated on the right side of each spectrum. Spectra in blue show events that scored over the threshold value on the MCR model. Spectra in red did not score above the threshold on the MCR model. Dashed line at 593 cm^{-1}.

Equation 1

The equation for flux to a surface is defined as: $J = 1.47(\frac{DA}{h})^{2/3}CQ^{1/3}$ where J is the flux through the flow cell, D is the diffusion coefficient of the analyte, A is the area of the surface, or

in this case the area of the laser spot, h is the height of the channel, C is analyte concentration, and Q is volumetric flow rate.[11] For our system, the terms are defined as follows. $D = 10^{-5}$ cm^2/s, $A = (0.6\ \mu m)^2\Pi$, h = 500µm, C = 500 pM, and Q = 1 µL/min. This equates to about 200 molecules/s. For a 10 ms acquisition time, that leads to 2 molecules/acquisition.

References

1. S. M. Asiala and Z. D. Schultz, *Analyst*, 2011, **136**, 4472-4479.
2. A. Nguyen and Z. D. Schultz, *Analyst*, 2016, **141**, 3630-3635.
3. P. Negri, K. T. Jacobs, O. O. Dada and Z. D. Schultz, *Analytical Chemistry*, 2013, **85**, 10159-10166.
4. H. C. Schorr and Z. D. Schultz, *Analyst*, 2023, **148**, 2035-2044.
5. M. R. Bailey, A. M. Pentecost, A. Selimovic, R. S. Martin and Z. D. Schultz, *Analytical Chemistry*, 2015, **87**, 4347-4355.
6. K. T. Jacobs and Z. D. Schultz, *Analytical Chemistry*, 2015, **87**, 8090-8095.
7. M. Nasir, D. R. Mott, M. J. Kennedy, J. P. Golden and F. S. Ligler, *Microfluidics and Nanofluidics*, 2011, **11**, 119-128.
8. J. P. Golden, G. A. Justin, M. Nasir and F. S. Ligler, *Anal Bioanal Chem*, 2012, **402**, 325-335.
9. T. O'Haver, *Journal*, 2022.
10. N. N. Brandt, O. O. Brovko, A. Y. Chikishev and O. D. Paraschuk, *Appl Spectrosc*, 2006, **60**, 288-293.
11. S. Sjoelander and C. Urbaniczky, *Analytical Chemistry*, 1991, **63**, 2338-2345.
